# Measure cross-sectoral structural similarities from financial networks

**DOI:** 10.1038/s41598-023-34034-w

**Published:** 2023-05-02

**Authors:** M. Boersma, J. Wolsink, S. Sourabh, L. A. Hoogduin, D. Kandhai

**Affiliations:** 1 Computational Science Lab, University of Amsterdam, Amsterdam, The Netherlands; 2 KPMG, Amstelveen, The Netherlands; 3 KPMG Global Solutions Group, Berlin, Germany

**Keywords:** Applied mathematics, Computational science

## Abstract

Auditing is a multi-billion dollar market, with auditors assessing the trustworthiness of financial data, contributing to financial stability in a more interconnected and faster-changing world. We measure cross-sectoral structural similarities between firms using microscopic real-world transaction data. We derive network representations of companies from their transaction datasets, and we compute an embedding vector for each network. Our approach is based on the analysis of 300+ real transaction datasets that provide auditors with relevant insights. We detect significant changes in bookkeeping structure and the similarity between clients. For various tasks, we obtain good classification accuracy. Moreover, closely related companies are near in the embedding space while different industries are further apart suggesting that the measure captures relevant aspects. Besides the direct applications in computational audit, we expect this approach to be of use at multiple scales, from firms to countries, potentially elucidating structural risks at a broader scale.

## Introduction

Audit is a 217 billion-dollar market which is projected to grow to 287 billion dollar by 2027^1^. An audit is a service where a third party (the auditor) assesses whether financial information published by a company is presented truthfully. The truthfulness of financial information is crucial, but existing methods to audit data are mostly manual in nature, and even nowadays, few algorithmic audit procedures exist. This is surprising because auditors have access to the data of the companies they audit, which presents them with a unique opportunity to understand companies through the lens of algorithms and data^2^. The audited information is then used by investors, creditors, traders, and lenders on a daily basis to make decisions. The impact of misstated financial information can have devastating effects, as the 2020 WireCard fraud case shows: 1.9 billion dollars went missing at the payment processor, resulting in its bankruptcy^3^. Moreover, aggregating the data of multiple companies provides an opportunity to understand our economy better.

Traditionally, central institutions such as the European Central Bank collect financial information to obtain a view of the state of the economy. Audits assessing the truthfulness of financial information are conducted before companies can share it. Such audits can take months to complete. Timely insights, however, are crucial in response to a crisis: a timely assessment of the economic distress proved essential during the recent COVID-19 pandemic. Almost ironically, the majority of algorithms applied in audit require financial information as input while often those numbers are unknown or at least uncertain, more so, non-trivial to construct from detailed transaction data. For example, fraud scores such as M-score^4^ and F-score^5^ all require aggregated financial figures as inputs that need to be constructed from transaction data. But a human expert is required to annotate the data, and therefore approaches such as these are hard to automate. Just recently new algorithms were proposed that aim to provide useful audit analysis without human intervention^6–10^. The challenge, therefore, is this: how can we use the transaction data to understand the company and at the same time avoid human annotation to answer relevant questions?

In general, to understand an object, we often compare it to other, similar objects. Audit is no exception to this. In our research, we want to compare a new client with peers, with peers being similar companies. This helps the auditors to assess risks early on in the audit process. Traditional ways to compare companies are, for example, ratio analysis, but this method requires a human-in-the-loop to annotate and structure the data.

Other domains facing comparable issues found similar solutions, by identifying good mathematical representations of the problem they studied. For example, scientists represented molecules and genes as networks that enable the use of advanced deep learning methods^11,12^ with the ultimate goal of finding good mathematical representations or combining human-expert knowledge with deep learning methods^11^. Similarly, economic phenomena have been studied as a network representation, providing a more realistic perspective compared to existing models^13–17^. Networks play a central role to study complex systems^18^. Once a domain identifies a good representation, applications are almost limitless, as shown by the sheer volume of research papers in this domain.

Our research approach is to apply the above concept to companies. Instead of classifying molecules as soluble or toxic, we want to work towards methods that can classify unhealthy companies, detect unexpected changes in their structure and find the most similar companies to compare them with. To do so, we propose creating an embedding space where each item is a mathematical representation of a company derived from transaction data.

**Figure 1:**
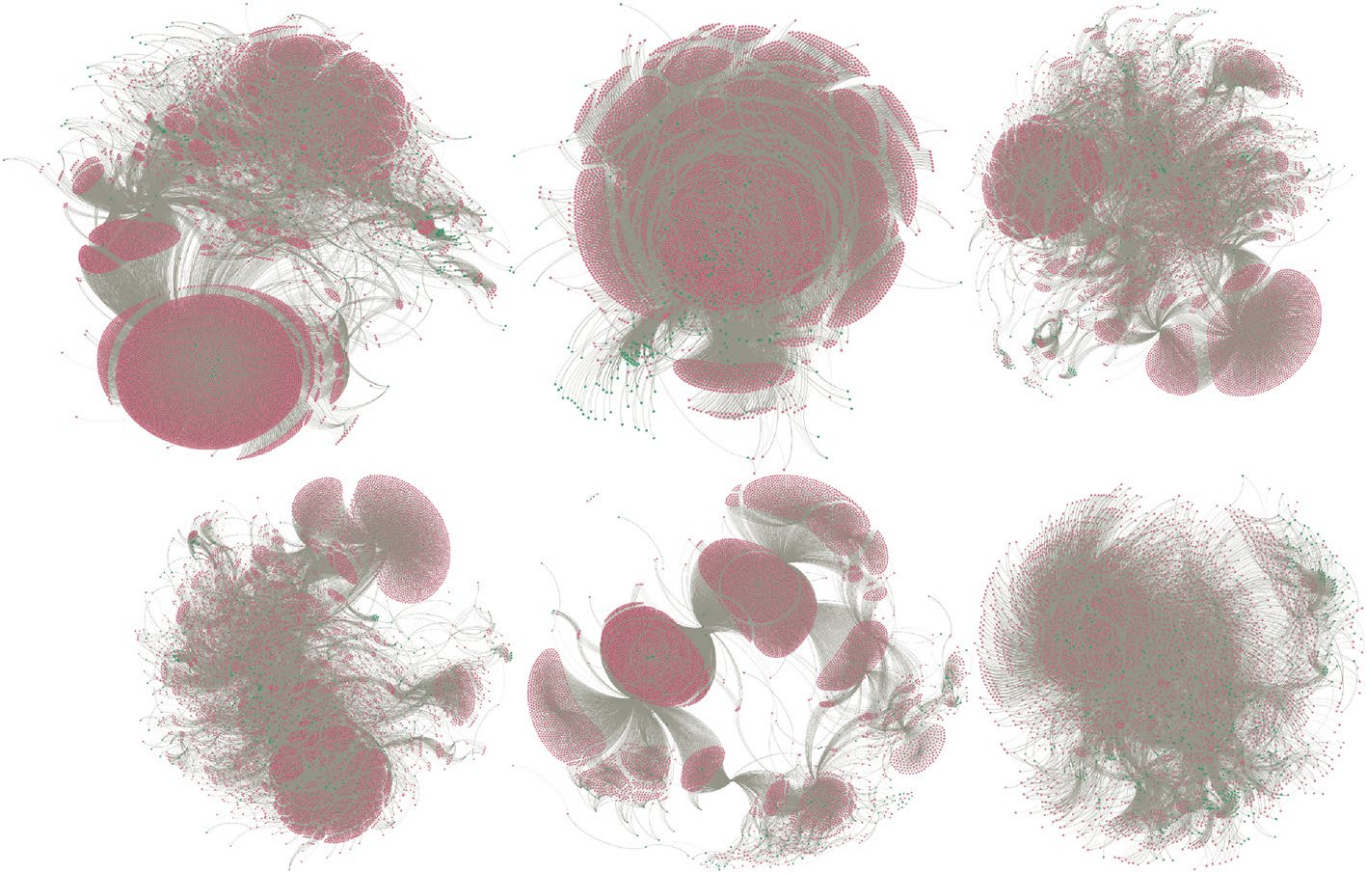
A sample of 6 networks from the 300+ dataset. We rendered these networks using the Fruchterman–Reingold method^40^. A visual inspection makes it clear that these networks are structured differently, and in some cases intriguing patterns appear like the clusters we observe in the networks above.

The transaction data of a company provide a comprehensive view of its money flows^6,19^. Assessing the overall complexity of the money flows is valuable. Even more important is understanding whether this complexity is similar over consecutive years and with respect to peers, as this information helps the auditor to assess the audit risks: large differences could indicate a difference in audit risk. Recent publications show that such transaction data can be represented as a financial statements network^19^ that provides a detailed view of the financial complexity of a company without a human annotator being required (see Fig. 1 for examples). As a result, the question of whether two companies are similar becomes a question as to whether their network representations are similar. Network similarity is an emerging machine learning research field that has already resulted in useful applications in domains like life sciences. Therefore, we propose representing a company as a vector of network similarities between the network of that company and other reference companies. This results in a vector representation (embedding) of a company. The embedding space which contains all vector representations is then a mathematical description of the companies in the economy.

To validate whether the outcome leads to useful vector representations, we performed a classification task. We expect companies from the same industry to have a shared audit approach and risk profile. Those companies are more similar than companies from different industries and this should allow us to identify good classifiers to separate the industries. Moreover, we investigated the obtained embeddings by studying the closest neighbors and understanding their similarities. And finally, we measured the similarity between two consecutive years and detected significant modifications in the underlying transaction data.

### Network similarity literature review

In life sciences, network similarity is used to predict useful properties of molecules. The molecules are presented as network structures. For example, in the quest to design a soluble molecule, it is necessary to predict, before production, whether a newly designed molecule is soluble^20^. As a consequence, multiple network datasets for which the molecule properties are known have been created, including PTC (predict carcinogenicity for rats), ENZYMES (600 protein tertiary structures) and more^21^. The datasets are used to create new algorithms that predict the properties from the network structure. Moreover, these datasets are also often used as benchmarks to design graph classification algorithms. The research area of graph classification is broader than the life sciences domain, and includes for example predictions of the genre based on a network of actors that appear in the same movie (IMDB-Binary movie dataset). In our research, we have opted for a similar approach. We took a network representation obtained from a company’s transaction data^19^ and used it for a variety of down-stream methods like determining their similarity. The research of graph classification can be categorized in (1) representation learning methods and (2) kernels methods. These methods learn or define a feature vector of a graph that accurately captures the relevant aspects of a graph. The vectors obtained are used in downstream tasks such as graph classification with an Support Vector Machine (SVM) algorithm.

Representation learning is often done with a neural network, where the vector representation problem is formulated as a learning problem. For example: obtain a node vector representation by using a neural network to predict the neighbours of a node in the network^22^. The weights of the first layer of the network—the embedding layer—is used as a vector representation for the input node. Early work such as DeepWalk^22^ and Graph Convolutional Networks (GCN)^23^ formulates similar learning problems to learn an embedding (feature) vector for graph components, the nodes and edges. More recent work focuses on learning a representation for the graph as a whole^24–27^. Although formulating graph representation as a learning problem has as an advantage in that it will learn features that describe the graph, the resulting vector representations are difficult to interpret and explain. Clarity is essential for audits where the information is used for analytical purposes—preferably ones that can easily be understood.

By contrast, kernel methods do not learn feature vectors but define them, whether explicitly or implicitly using the kernel trick. Most proposed kernels in the context of graphs are special cases of the general R-convolutional kernels^28^. Instead of comparing an object as a whole, it is described as a collection of parts, and those parts are then compared. For example, kernels that count the number of Sub-tree patterns^29^, Cyclic patterns^30^, Graphlets (motifs)^31^, Shortest paths^32^, Random walks^33,34^, and Propagated labels [Weisfeiler–Lehman (WL)]^35^. Each of the kernels transforms the graph object into a feature of, for example, counts of a particular pattern. When two graphs have similar feature vectors, the distance (Euclidian or Cosine) will be small and therefore the graphs are similar.

As an alternative to defining parts and patterns, any object can be described as a vector of dissimilarities or similarities with respect to other objects^36^. This idea of a dissimilarity vector can also be applied to graph objects^27,37,38^ and is attractive for auditors because it yields a natural interpretation. We describe an object as being similar to other objects. For example, if we take Exxon Mobil (oil and gas), we state that it is similar to Shell (oil and gas) and dissimilar to J.P. Morgan (bank). We still have to determine how to measure the similarity between their mathematical representations—a financial statements network of the company’s transaction data.

It is interesting to note that a similarity measure can be learned and defined. Deep Divergence Graph Kernels (DDGK)^27^, a neural network approach, learns whether two graphs are similar or dissimilar. Graphs are encoded by training the neural network that takes a node as input and learns to predict its neighbours, essentially encoding the structure of the graph into the model. The trained neural network of graph A is used to make predictions in graph B; if this results in good predictions then the learned encoder encodes the properties in both graph A and B properly and therefore graph A and B must be similar. The prediction errors are a proxy for the similarity between graph A and B. However, training the DDGK neural networks to encode a graph’s properties is computationally expensive because a neural network needs to be trained per graph. An alternative method that does not require training a neural network, described by Togninalli et al.^37^, uses the Weisfeiler–Lehman algorithm to propagate labels in the network—this implicitly captures relevant properties like node degree and node labels—and measures the distance between the distribution of the propagated node labels between two graphs using the Wasserstein distance. When two graphs have similar structures, their propagation is similar and therefore the distance between their distributions is small. As a result, graphs with small Wasserstein distance are similar. Both methods do not require us to define features for the graphs.

### Aim and contribution

In our research, we opted for an automated procedure that uses detailed transaction data to create a vector representation of the company. The vector representations can be used for a variety of tasks, for example, finding the most similar company and monitoring structural changes in the accounting system. We contributed the following:

- we analyzed 300+ datasets from real companies;
- we defined a similarity vector as a mathematical representation of a company measured through its network similarities;
- we created an embedding space based on the vector representations that contains hundreds of companies that can be used for a variety of tasks;
- we showed a variety of auditing applications that use the embedding space.

**Figure 2:**
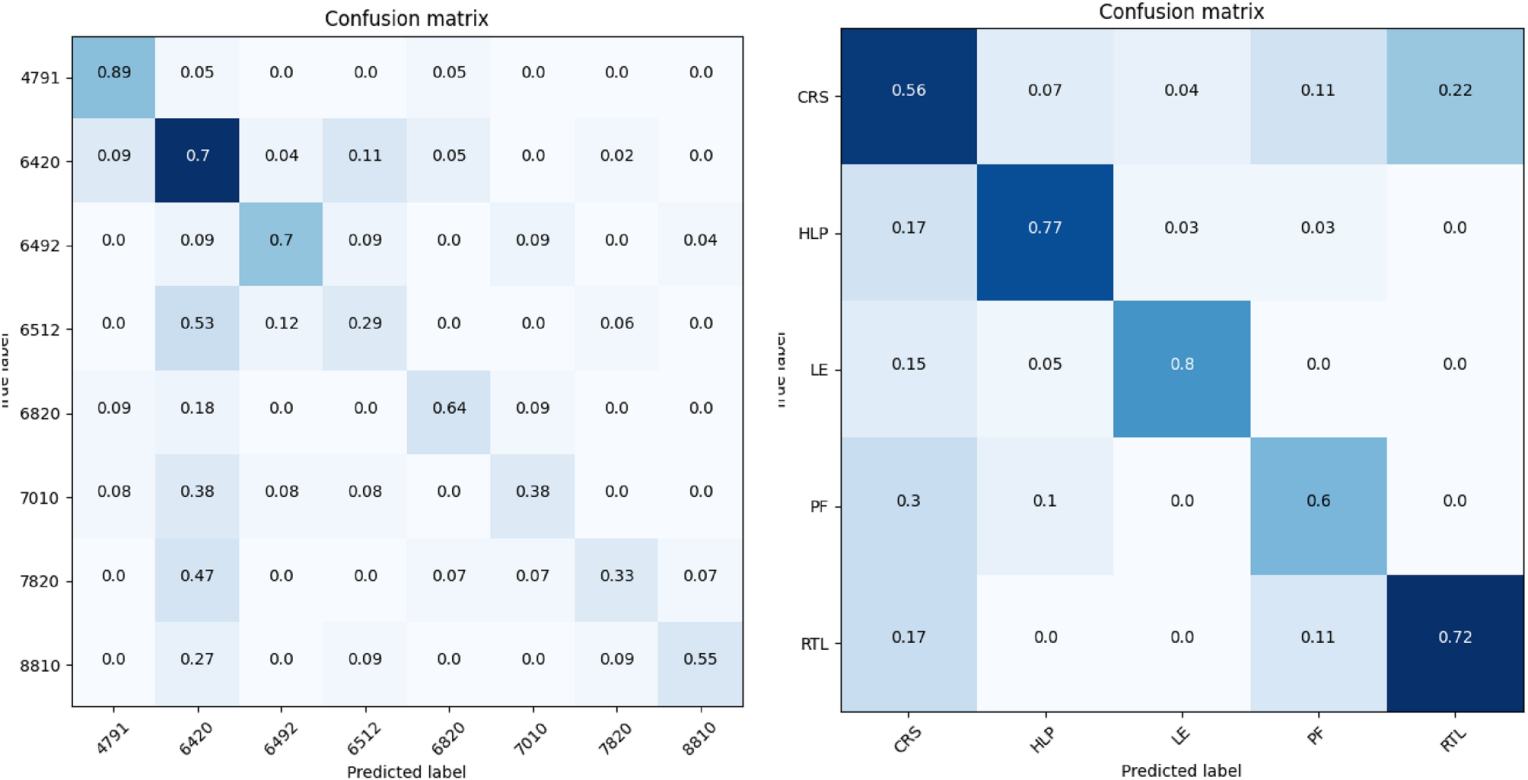
The confusion matrix with on the left the confusion matrix of the NACE industry codes and on the right the confusion matrix for the expert’s labels Detailed Industry. Note that these confusion matrices for NACE have at least 10 items per clas, and the Detailed industry 20 items per class. We have the following industries: General manufacturing (CRS), Healthcare (HLP), Energy (incl. oil and gas) (LE), Publishing (PF) and Retail (RTL), Activities of holding companies (6420), Other credit granting (6492), Online Retail sale via mail order houses or via Internet (4791), Non-life insurance (6512), Rental and operating of own or leased real estate (6820), Activities of head offices (7010), Temporary employment agency activities (7820), Social work activities without accommodation for the elderly and disabled (8810).

Our aim is to test the hypothesis that we can detect similarity between companies belonging to the same industry type, based on their network representation from financial transaction data. With this contribution we provide a novel approach to do an automated more comprehensive risk assessment, paving the way for automated continuous audit procedures (Fig. 2).

**Table 1:** The average tenfold cross validation classification accuracy and standard deviation for the various labelled datasets using a random selection of reference networks.

Industry (NACE)	Industry	Detailed industry	International	Large balances
61.16 ± 4.2	64.89 ± 10.56	74.83 ± 11.46	77.29 ± 5.01	76.65 ± 7.41

## Results

We hypothesize that we can determine the similarity between companies based on their network representation using financial transaction data. To do so, we collected the transaction data of 300+ companies, and we constructed various categorizations, such as the industry to which each company belongs and whether it is domestic or not. We aim to test whether we can learn such categorizations from the network representation. In this section, we explain the main results, and for details on the dataset, how we construct the network, how we propagate values through the network, and how we exactly construct a similarity vector representation for each company we refer to the “Methods” section.

**Figure 3:**
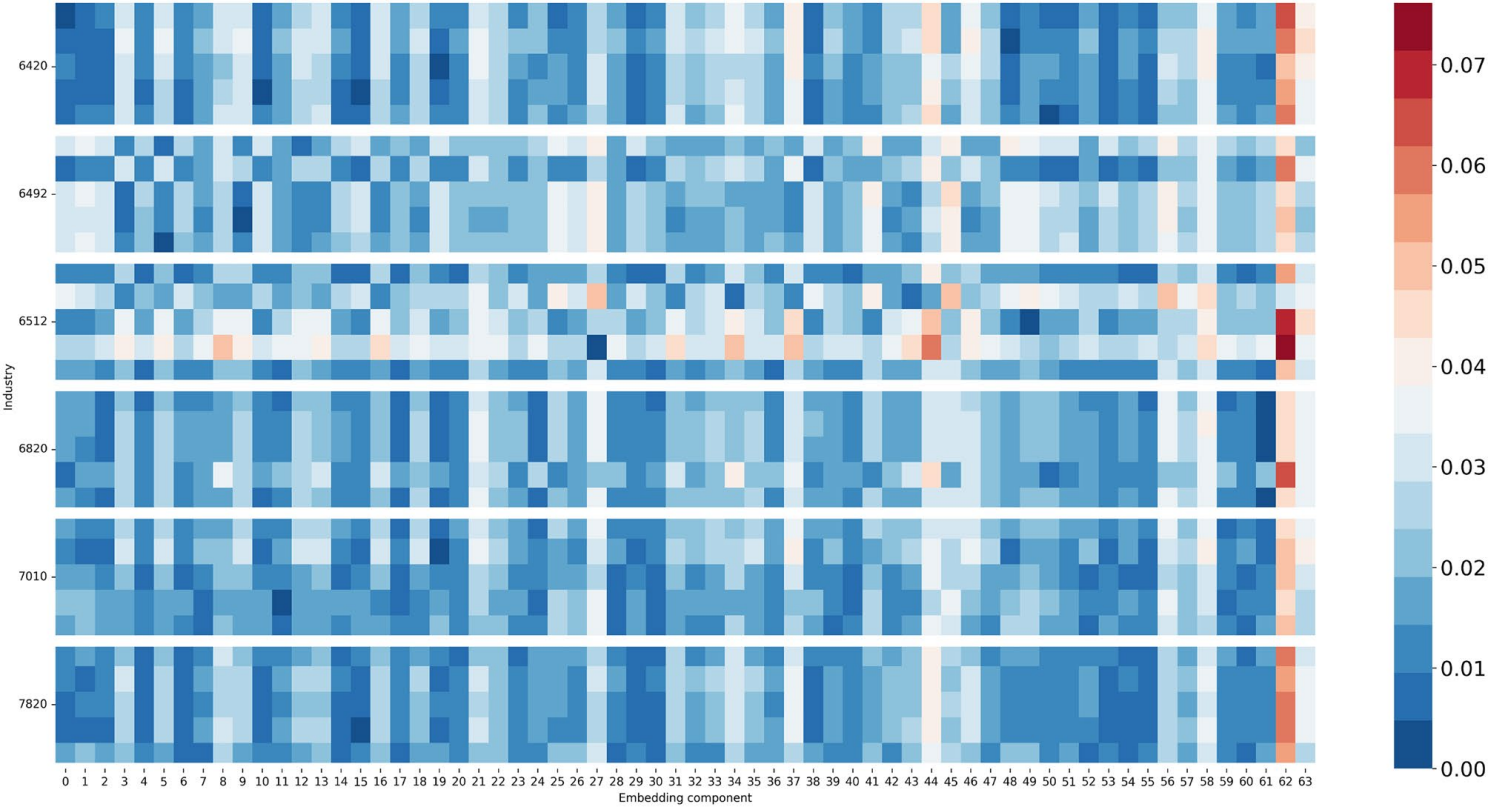
Each row represents a company which we express as being similar or dissimilar to the reference companies (x-axis components measure the similarity). The colours represent the normalized vector values. We grouped the companies per NACE industry: Activities of holding companies (6420), Other credit granting (6492), Online Retail sale via mail order houses or via Internet (4791), Non-life insurance (6512), Rental and operating of own or leased real estate (6820), Activities of head offices (7010), Temporary employment agency activities (7820). The figure shows vectors from different companies in the same industry.

To create a vector representation for each company based on its transaction data, we first convert the data into a target network, and then measure the similarity of the target network to a set of *k* reference networks. As a result, we represent each company as a vector with *k* features. We want to set *k* such that the vector’s features encode sufficient information to perform well on downstream tasks. To measure the similarity, we create two histograms of the monetary values recorded in each node of the target and reference network, we then propagate the values through the network and repeat the first step. After repeating this for *h*-steps, we measure the Wasserstein distance between all histograms of the target and reference network. This results in a vector of size *k* that describes the target network. We found that using this vector representation improved our ability to classify a company’s industry type. Figure 1 shows 6 real networks, Fig. 2 shows the confusion matrix, and Fig. 3 shows the vectors we obtain. With these vectors, we obtain 61% prediction accuracy for the NACE classes and 75% accuracy for the Detailed Industry using an SVM classifier (see Tables 1, 2, see for details about the classes “Company dataset”).

In Fig. 2 we show the confusion matrix for the NACE and Detailed Industry classes. We normalize the confusion matrix on a per-row basis to provide us with insights about how the predicted outcomes are distributed among the various classes. It is noteworthy that the true positive scores range between 29 and 89% for the NACE classes, and between 56 and 80% for the Detailed Industry classes. The results in the 6420 (left figure) and CRS (right figure) columns imply that the algorithm has difficulty distinguishing* Activities of holding companies* from others and *General Manufacturing* companies from others, which could be explained by the fact that both classes are the largest in the dataset, resulting in bias towards this class. Nonetheless, the algorithm can distinguish various industry types such as *Activities of holding companies (6420)*, *Other credit granting (6492)*, *Online Retail sale via mail order houses or via Internet (4791)*, and *Rental and operating of own or leased real estate (6820)*. For the Detailed industry (expert) classes it can distinguish betweeen *Healthcare (HLP)*, *Energy (LE)*, and *Retail (RTL)*. A possible explanation for the difference in performance between the NACE classification and the expert classification is that many companies are classified as holdings (6420) whereas the expert would classify them differently based on audit approach, e.g., a holding of a retail company is classified as retail by the expert.

To ensure the effectiveness of our algorithm, we conducted various tests and evaluations. This includes analyzing the impact of the selection method for the reference networks, and the impact of the hyper-parameters *k* and *h*. Moreover, we compared our results to those of the baseline that assigns all items to the largest class. We found that selecting *k* reference networks randomly worked well (see Supplementary Materials (SM): Table S4) and that using $k=64, 128$ and $h=7$ provided good results (see SM: sensitivity analysis). In addition, we observe that the weights and direction of the edges, as well as scaling the nodes’ features, improved our classification performance, see Tables 3 and 4 respectively. We compared our results with a naive classifier which always predicts the largest class. We used a *t* test^39^ with two hypothesis: a null hypothesis that states that the mean accuracy of our model and the naive model are equal, and the alternative that states that they are unequal. We obtain *t* test *p* values: 0.00, 0.00, 0.00, 0.01 and 0.00 for each column in Table 1 respectively. For a *p* value lower than 0.05, we reject the null hypothesis and conclude that the means are significantly different, and a more detailed inspection revealed that we obtained an improvement. For the domestic versus non-domestic (international) and large versus small balances (large balances) classification task, we outperformed the naive baseline with a smaller margin. In addition, when we queried companies’ nearest neighbors, we often found companies showing a clear similarity to the selected company. Moreover, we measured the similarity between consecutive years of a company and we were able to detect whether significant structural changes occurred in the transaction data (see SM: extended evaluation of the company dataset).

These results, from a quantitative and qualitative aspect, suggest that we found a good vector representation that can be used by auditors to understand their new client during the audit planning phase. For validation purposes, we applied the algorithm not only to the company dataset but also to synthetic datasets and public datasets (see SM: Synthetic networks, Public networks) that are often used as benchmarks, and we obtained results at par with other state-of-the-art algorithms (see SM Table S2 and S3).

The remainder is organized as follows, we discuss the vector representation in more detail. In the “Discussion” section, we elaborate on our conclusions and suggest future work, and in the “Methods” section we included the necessary details to reproduce the research.

### Vector representation

In contrast to the work of Togninalli et al.^37^, we used a subset of reference graphs resulting in a vector representation. In the subsections below we discuss our investigation of the various dimensions of this choice. First, we discuss the impact of a subset selection. Then we examine the impact of the financial network structure itself on the classification task by removing elements from the network like node attributes, edges, and edge weights. And, finally, we investigate the impact of the classification method by using the AutoML framework^41^ as an alternative to the SVM classifier. The AutoML framework searches for the best classification model in a given time frame. Each experiment setting was repeated 10 times to obtain mean results and standard deviations.

#### The impact of the subset selection

We assumed that selecting a good subset of reference nodes would improve the performance. We, therefore, evaluated two methods of selecting reference graphs: (1) a k-medoids algorithm and (2) the random selection algorithm. The k-medoids selects *k* reference networks from clusters that are dissimilar from one another to increase the expressiveness of the similarity vector which should result in better predictive performance. Table 2 shows the classification accuracy for the two selection methods. First of all, on public datasets, using a subset instead of the whole set yields similar performance (see SM: Table S2 and S3 for results on the public datasets). Moreover, Table 2 shows for the company dataset that using a random selection yields similar results as the k-medoids selection while the k-medoids is computationally more expensive.

**Table 2:** The average tenfold cross validation classification accuracy and standard deviation for the various labeled datasets and selection method.

Model	Industry (NACE)	Industry	Detailed industry	International	Large balances
K-mediods	61.72 ± 5	64.65 ± 9.53	72.25 ± 15.26	77.59 ± 6.38	77.58 ± 6.53
Random	61.16 ± 4.2	64.89 ± 10.56	74.83 ± 11.46	77.29 ± 5.01	76.65 ± 7.41

The accuracy scores are similar while the computational complexity of k-mediods is higher.

#### Network structure

We assumed that taking into account the network structure using the propagation steps would improve the classification accuracy. Therefore, we investigated the impact on the performance of the network structure, node attributes, and edge attributes. Table 3 shows the accuracy scores for the company dataset. The network with all its features—weighted edges and node labels—results almost always in the highest classification scores. However, making the edges unweighted while propagating values resulted in higher classification scores for the industry and large balances classification tasks. Removing the edges from the network resulted in significantly lower scores because the propagation step could not be applied. When we removed the node labels—the monetary value—from the network, the accuracy scores decreased even further. An explanation for the changes in prediction accuracy is that the network attributes influence the propagation of monetary value in the network. The propagation step explicitly takes into account the edge’s weights and the node’s value (see “Methods” section). Consequently, we obtain different histograms that we use to calculate the similarity. Thus, we capture the subtle difference in a company’s financial structure in the similarity measure. For example, consider two networks that only have different edge weights. Different edge weights could be caused by subtle differences in the same business process, for example, they both sell goods but with different tax rates. This ultimately results in a slightly different propagation step, and we can measure that difference in the Wasserstein distance calculation. Not surprisingly, if we consider the most detailed classification task, the Detailed Industry labels, the accuracy drops when we do not consider properties such as edge weights. The results suggest that the details of the network such as edge weights become more important. The details enable us to capture the subtle difference that defines a class. As a result, the prediction accuracy of the classifier increases. Moreover, we investigated the impact of the node features: the monetary volume in each node. Table 4 shows that scaling the node values by taking the square root or the log increases the classification accuracy.

**Table 3:** The average tenfold cross-validation classification accuracy and standard deviation for the companies dataset for various network construction methods.

Network type	Industry (NACE)	Industry	Detailed industry	International	Large balances
Edges removed	58.69 ± 7	60.88 ± 12.07	66.25 ± 14.35	75.64 ± 6.17	72.12 ± 7.62
Node attributes removed	43 ± 0	56.1 ± 5.99	54.38 ± 6.94	73.41 ± 6.15	68.63 ± 7.10
Unweighted edges	58.01 ± 12.56	66.98 ± 9.34	70.25 ± 12.91	75.35 ± 7.50	83.03 ± 6.16

The results show that edges and node attributes increase the accuracy significantly while the edge weights only improve the results for the detailed industry. See Table 2 for the performance with all network attributes.

**Table 4:** The average tenfold cross-validation classification accuracy and standard deviation for the companies dataset, the results show that scaling the node attributes (monetary value) improves the classification accuracy.

Dataset	Industry (NACE)	Industry	Detailed industry	International	Large balances
Companies	52.48 ± 2.1	50.45 ± 2.72	51.75 ± 9.68	72.44 ± 2.06	68.61 ± 5.22
Companies (sqrt)	53.11 ± 3.28	56.48 ± 11.42	62.91 ± 13.03	73.01 ± 2.47	83.40 ± 7.17
Companies (log)	61.16 ± 4.2	64.65 ± 9.53	72.25 ± 15.26	77.59 ± 6.38	77.58 ± 6.53

#### Classification algorithms

We investigated the influence of the classification methods. For the companies dataset, we consistently obtained a lower score for the AutoML model compared to the SVM classifier. For the public datasets, SVM almost always outperformed the AutoML classifier but for the IMDB-M dataset we achieved higher accuracy than any other model (see SM Table S2 and S3).

## Discussion

In our research, we focused on identifying an efficient data-driven measure of similarity between companies. We found that the distance in the vector space resembles the similarity between companies. To do so, we measured the similarity between 300+ companies by analyzing their transaction data, applying an automated process. A degree of similarity can serve as a starting point for new audits, by copying a specific audit approach of similar companies. Moreover, a degree of similarity can also act as a warning signal for existing audits, because potential risks show up through unexpected differences. We show that we can measure the similarity between companies by transforming their transaction data into a network and measuring the similarities between networks. Traditional methods used to compare companies, such as financial ratios, often require a deep understanding of the client’s accounting system that records the transaction data, and a manual effort to create financial statements or ratios that can be compared between clients (as an example: some financial records are not in English, making comparison non-trivial). To the best of our knowledge, this is the first algorithm that processes the transaction data in an automated manner to determine whether clients are similar.

We show the impact of various aspects of the algorithm on the performance, including the selection of reference networks, the network structure itself and the impact of the classification algorithm. Our results suggest that the similarity representation is a useful representation of the transaction data that can be applied to better understand new audit clients. Moreover, we show that our proposed modifications of the work of Togninalli et al.^37^ score well on public benchmark datasets (see SM: WWL modifications and Table S2 and S3) and in some cases achieve even higher scores.

From a qualitative perspective, we found interesting clusters in our representation. For a retailer active in Europe, we found two clusters representing the retailer’s activities: one cluster in Europe and the other in Scandinavia. Moreover, we selected an insurance company and found other insurance companies in the k-nearest neighbours list based on the Euclidean distance (see SM: Extended evaluation of the company dataset for more in-depth analysis and visuals). Figure 3 visualizes that the vector representations within an industry are more similar than between industries. This suggests that networks, as displayed in Fig. 1, are more similar because they have characteristics in common.

In addition, we detected modified transaction data from the same company in consecutive years. We selected three companies and measured their natural difference in similarity between the 2019 and 2020 vector representation. We then modified the 2020 transaction data by removing 50% of the journal entries and inserting 50% of the journal entries of another company. We could see a decrease in the cosine similarity score after the modification (see SM: manipulation detection). This gives auditors a helpful insight in understanding whether significant changes occurred in the complexity of the transaction data in consecutive years to help them identify a potential change in audit risk. Moreover, we used a subset of reference companies to represent each company as a similarity vector which results in higher computational efficiency. The results are approximately on par with the Wasserstein Weisfeiler Lehman (WWL) algorithm^37^, this while the complexity of the WWL algorithm is $O(N^2)$ with *N* as the number of networks (WWL computes a similarity matrix for each pair of networks) whereas we take $k<< N$ as a reference set of networks such that the complexity is *O*(*Nk*) which results in a significant improvement.

For future research, we would like to focus on whether it is possible to detect which aspect of the network is different when comparing two networks. In our current research, the algorithm can identify the similarity between two networks, but it does not point to a substructure of the network that is different with respect to the other network. This would be a useful addition because, if an unexpected dissimilarity shows up, the auditor wants to know which parts are dissimilar. Moreover, we recommend to research whether these techniques are capable of detecting fraud by injecting fraudulent transactions in the dataset. More specifically, the sensitivity with respect to small modifications by adding, modifying or removing structures in the network caused by fraudulent behavior. In addition, it would be interesting to collect more longitudinal data and study how the network structure responds to important events like the financial crisis of 2008 or the COVID pandemic.

## Methods

We use the WWL algorithm proposed by Togninalli et al.^37^ to measure the similarity between companies (Note that we made small modifications to improve the WWL algorithm, see SM: WWL modifications for details.). To compute the similarity, we first transform the transaction data into a network representation and use the network representation to propagate monetary value through the network using the continuous Weisfeiler–Lehman algorithm^37^. As a result, we obtain different distributions of monetary value among the nodes in the network. We use the Wasserstein distance metric to measure how similar two networks are based on their propagation histograms. Finally, we represent each network as a vector of similarity measurements with respect to a set of reference networks.

### Transaction data to network representation

**Figure 4:**
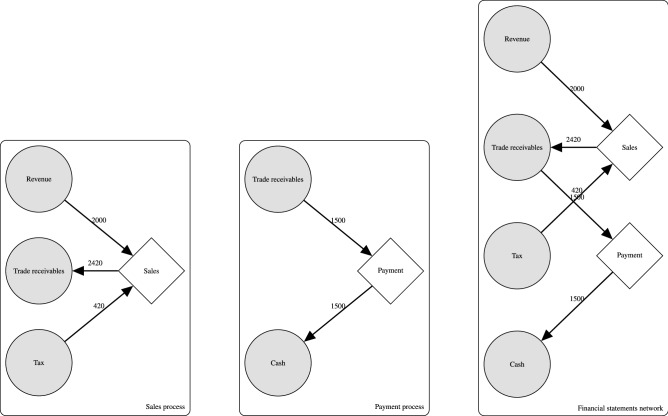
Example schematic diagram of the business process depicted in Table 5.

We explain what a company’s transaction data is about, and how to transform this into a network representation. Large companies are often obligated to share their performance in an annual report. This report contains the financial statements which summarize the company’s financial performance. The company’s activities that affect its financial performance are recorded in an accounting system, for example, goods sold or materials purchased are recorded in transaction records. Table 5 shows two sample transaction records—the journal entries. The journal entry with ID 1 represents a sales activity, and the journal entry with ID 2 represents the payment of the sales invoice. The number of journal entries in a year depends on the size and complexity of a company but can easily amount to tens of thousands of entries for small companies and even millions of journal entries for large companies. As part of our research, we transformed all journal entries into a network representation for further analysis^19^. This resulted in a bipartite *financial statements network*^19^ with *financial account* nodes and *business process* nodes. The financial account nodes are often (in aggregate) represented in the financial statements, for example, the revenue account, the cash account and more. The business process nodes represent the unique journal entry structures we encounter in the journal entry dataset. For example, when we have another sales entry similar to ID 1 in Table 5, we say that we have one process (sales) but different amounts. When another sales record similar to ID 1 includes another line with a discount we say that this is another unique process (sales with discount). More formally, we can search for the set of unique business processes as follows:^19^

1 $$\begin{aligned} B: \sum ^m_{i=1} \alpha _{i} A_{i} \Rightarrow \sum ^n_{j=1} \beta _{j} A_{j} \end{aligned}$$

where *m*, *n* are the number of credit and debit financial accounts in the journal entry. $A_i$ is a financial account and $\alpha _i$ is the percentage credited with respect to the total credit amount in the journal entry, $A_j$ are the financial accounts debited and $\beta _j$ is the percentage debited similar to the credit coefficient. For example, for the journal entry with ID 1 in Table 5, we have $\alpha _1 = 2000/2420 = 0.83, \alpha _2 = 420 / 2420 = 0.17$ and $\beta _1 = 1$. In Fig. 4 we show the network representation of each journal entry from Table 5. We combined all these journal entry networks to create the financial statements network (see Fig. 1 for real-world example networks). Once we had multiple networks of various companies, we described each company as being similar with respect to other networks.

**Table 5:** Transaction with ID 1 is a Sales transaction and transaction with ID 2 is a Payment transaction.

ID	Name	Journal	Date	Debit	Credit
1	Trade receivables	Sales ledger	1-1-2019	2420	
1	Revenue	Sales ledger	1-1-2019		2000
1	Tax	Sales ledger	1-1-2019		420
2	Cash	Journal ledger	2-1-2019	1500	
2	Trade receivables	Journal ledger	2-1-2019		1500

### Company dataset

Our company dataset consists of 300+ companies mostly from Europe. For each company we obtained all the journal entry data (transaction data) for the year 2020 and for a limited set of companies we obtained data for 2019 or 2018. We have up to 12 million journal entries for a single company in one year. The audit risk is determined by factors such as the balance sheet total, geographic location, and industry. We hypothesize, due to their financial network structure being different, that we can measure this from their vector representation. For the industry categorization we use two systems: an NACE/SBI classification system, and an expert classification. The NACE/SBI is a standardized framework categorized by an independent party—the Dutch Chamber of Commerce—which categorizes companies based on their economic activity, we refer to this as the Industry (NACE) classification. For example, the retail industry class could include companies like Walmart, and the banking class could include companies like Goldman Sachs. In the case of multiple NACE/SBI classes for a single company, we selected one NACE/SBI code as the industry. The expert classification is a categorization based on audit approach and risk profiles, we refer to this as Industry and Detailed Industry, where the Detailed industry is another more fine-grained classification. Note that, the expert classification systems may differ from the NACE/SBI classification as will be clear from the results. We assessed our proposed vector representation by testing whether we can find these categorizations in the vector space. We evaluated the prediction accuracy for the following categories:

1. Industry (NACE): 166 companies in 8 classes;
2. Industry: 246 companies in 4 classes;
3. Detailed Industry: 151 companies in 5 classes;
4. International: is a company domestic or not;
5. Large balances: whether a company has more or less than 50 million euros on theirf balance sheet at the beginning of the year, a proxy for the size of the company.

For the Industry (NACE) dataset, we selected classes with more than 20 members, to obtain a representative sample. This resulted in a subset of classes that have a decent number of items for our tenfold cross-validation experiment. For example, for the Detailed Industry, we obtained 10 random splits of 15 items with 3 expected items in each class. In brief, we have the following datasets for the classification tasks:

1. Industry (NACE): 166 companies in 8 classes;
2. Industry: 246 companies in 4 classes;
3. Detailed Industry: 151 companies in 5 classes;
4. International: 312 in 2 classes;
5. Large balances: 312 in 2 classes.

Notice that the Industry (expert), Industry (NACE), and Detailed Industry have 246, 166, and 151 companies instead of 312 because we select only classes with sufficient samples. In this dataset, we used 20 items per class for Industry and Detailed Industry, but for Industry (NACE) we use 10 samples per class because with 20 items we would only have 2 classes (see for detailed results for 10 and 20 items per class SM: Analysis of the confusion matrix). Moreover, the class imbalance is important, especially, because we use accuracy as a metric to evaluate our algorithm’s performance. For the Detailed Industry (expert), Industry (expert), and industry (NACE) datasets, we have a class imbalance of approximately 2:2:1:1:2 , 5:3:1:2, and 6:2:2:2:2:1:1:1 respectively.

### Wasserstein distance

**Figure 5:**
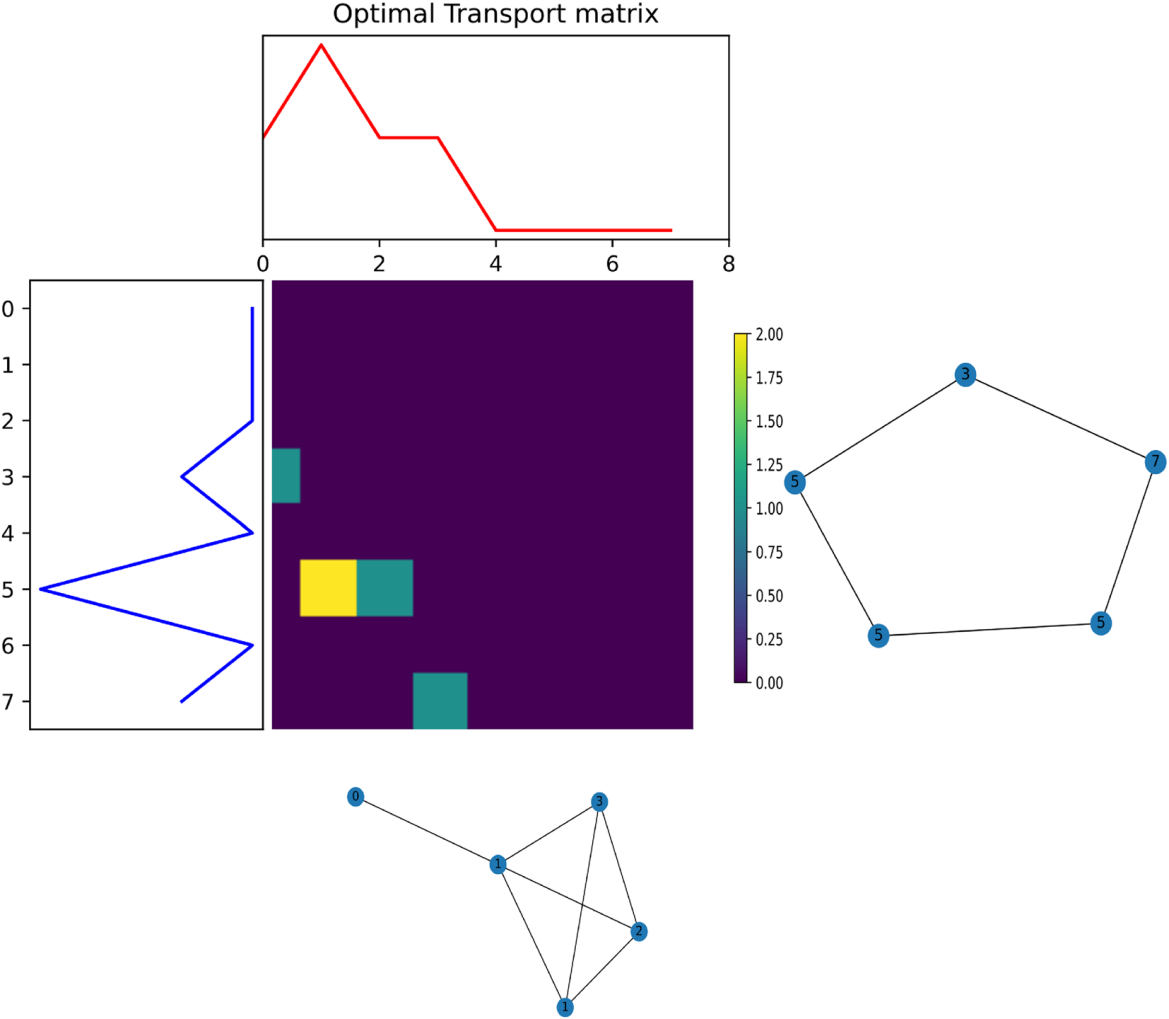
This figure shows the two networks, on the right and at the bottom, where each node in the network has a value. The red distribution at the top represents the distribution of the nodes’ values of the network at the bottom and the blue distribution represents the nodes’ value distribution of the network on the right. The matrix in the middle represents the optimal transport map of transporting the left distribution into the top distribution. The sum of all the transportation costs is the total Wasserstein distance as shown in Eq. 3.

The Wasserstein distance is used to measure similarity between two distributions. The Wasserstein distance measure is also described as the earth-mover problem: how much effort is required to transport the mass from one location to another. These transport maps and associated costs are used to find an optimal transportation map between two densities. The total costs of transportation are the summation of the masses you move times the distance it has to travel. In contrast, f-divergence measures, like the popular Kullback-Leibler divergence, do not take the distance between elements into account. Nonetheless, this is a relevant distance aspect. We apply the $L^p$-Wasserstein distance definition, where $p \in [1, \infty )$, from Togninalli et al.^37^:

2 $$\begin{aligned} W_p(\sigma , \mu ) = \bigg (\inf _{\upgamma \in \Gamma (\sigma , \mu )} \int d(x,y)^p d\upgamma (x, y) \bigg )^{\frac{1}{p}} \end{aligned}$$

where $\upgamma$ represents the transportation plans between marginal distributions $\sigma$ and $\mu$ (see Fig. 5 red and blue distribution) of all possible transportation plans $\Gamma$. A transportation plan describes how much probability mass from $\sigma$ moves to another location in $\mu$ with a distance *d*(*x*, *y*). For discrete settings this simplifies to a summation often represented as a Frobenius dot-product of the distance matrix M with the distances $d(x,x^{'})$ from the vectors $x \in X, x^{'} \in X^{'}$ and *P* as the transport matrix^37^:

3 $$\begin{aligned} W_1(X, X^{'}) = \min _{P \in \Gamma (X, X^{'})}<P, M> \end{aligned}$$

The vectors *X* and $X^{'}$ are the node value distributions. For example, to establish the amount of nodes we have with a particular value in the network, we obtain the vectors *X* and $X^{'}$ from networks *G* and $G^{'}$ as follows:

4 $$\begin{aligned} X_G^h = [a^h(v_1), \ldots , a^h(v_{n_g})]^T \end{aligned}$$

where $a^h(.)$ is defined in Eq. 5 and $v_i$ is a node from network *G*, and *h* is the number of propagations, and *T* is the transpose operation. From the vector $X_G^h$ we create a histogram that we use to obtain the optimal transport map. The matrix *M* can be computed as the Euclidean distance between the two vectors, and the optimal transport matrix is the matrix that minimizes the costs in Eq. 3. We use the Python Optimal Transport (POT) framework^42^ to calculate the optimal transport matrix.

For the financial statements networks in our research, in step $h=0$, we initialized the node values from the transaction data (see “Weisfeiler–Lehman continuous node attributes” section), in step $h=1$ we propagated the node values using Eq. 5. We repeated this up to the desired number of iterations. This resulted in a matrix where each row $i=1,\ldots ,h$ represents a vector $X_G^h$ and we use this matrix to compute a *h*-dimensional histogram for each network. For each network, we used this histogram to compute the Wasserstein distance between the two networks. Moreover, because the financial statements network is bipartite, we computed the Wasserstein distance between each partition of the network and the total Wasserstein distance is the sum between the two. That is, instead of computing the vector in Eq. 4 for all nodes, we only compute this vector for nodes from a single partition. We compute the Wasserstein distance between each partition to measure how similar their partitions are. Figure 5 contains an illustrative example of two (small) networks with a value distribution among their nodes (iteration step $h=0$) and the obtained distribution on the left and at the top. The heat map represents the optimal transport matrix *P*, a cell (*x*, *y*) in the heat map has an associated transport distance *d*(*x*, *y*) and the colour represents the amount of probability mass in transport. We use the Kantorovich relaxation^43^ which allows probability mass to split over multiple cells in the same row or column.

### Weisfeiler–Lehman continuous node attributes propagation

The propagation step propagates the continuous node values through the network structure as introduced by Togninalli et al.^37^. This is an extension of the classical Weisfeiler–Lehman kernel that propagates node labels and is known to capture network structures implicitly. In the financial statements networks we have continuous labels that represent the monetary amounts; note that we use a special initialization procedure because the financial statements networks are bipartite—the propagation steps remains similar to Togninalli et al.^37^. We propagated those values through the network (of known payment structures) and measured how the value distribution changed. We initialized the *business process* (BP) and *financial account* (FA) nodes as follows: for BP nodes we added up all the monetary amounts from the journal entries to obtain the total monetary flow of that business process. As we are interested in monetary flows, we initialized the FA nodes as zero. Moreover, instead of having directed edges, we modified the sign of the edge weight: negative for outgoing edges and positive for incoming edges. This enabled us to use the following propagation scheme from Togninalli et al.^37^:

5 $$\begin{aligned} a^{h+1}(v) = \frac{1}{2} \bigg (a^h(v) + \frac{1}{deg(v)}\sum _{u \in N(v)} w((v,u)) a^h(u) \bigg ) \end{aligned}$$

where $a^{h+1}$ is the new node value for node *v* where we average between the prior node value $a^{h}(v)$ and the weighted inflows and outflows *w*((*v*, *u*)) for neighbours *N*(*v*) of node *v* with *deg*(*v*) as the degree of node *v*.

After all iterations, we constructed a h-dimensional histogram and we computed the Wasserstein distance between the histograms. The combination of the value distribution with the propagation steps enabled us to detect whether initial value distributions are similar and whether the networks are wired in a similar manner. For example, densely connected networks converge faster to a peak distribution in contrast to sparsely connected networks. Both aspects helped us to assess the overall similarity between the networks.

### Similarity vector representation

One might wonder why we construct a vector of similarities instead a single similarity. The main reason is that a vector results in an expressive continuous representation of the network. The success of downstream tasks, such as the classification tasks proposed here, depends on a good representation of the network. A good representation should capture the relevant aspects that explain the variations in the data^44^. In contrast, a single similarity has limited expressiveness likely limiting the success of downstream tasks—two companies might be similar in one feature, but dissimilar in another. This while a (continuous) vector representation has higher expressiveness and could be used as input for a variety of classification and regression algorithms.

To define a representation for an object, we must learn or define its features. Objects are often described in terms of their characteristic features like colour, size and weight. They can, however, also be described relative to other objects: colour similar to that of an *Apple*, moves like a *Car* and goes as fast as a *Rabbit* describes a *green super car*. Likewise, to describe a company, we can say that Exxon Mobil (oil and gas) is similar to Shell (oil and gas) and dissimilar to J.P. Morgan (bank). For audit purposes, it is important that the representation is explainable. Therefore, we designed the features as a vector of similarities between other companies. Formally, we represent the company *x* as a vector $\phi _Y(x)$ of similarity measures (Wasserstein distance) with respect to a set of reference companies *Y*:

6 $$\begin{aligned} \phi _Y(x) = [W_1(x, y_1), W_1(x, y_2), \ldots , W_1(x, y_n)] \end{aligned}$$

where each $W_1(.,.)$ measures the Wasserstein distance of company *x* with respect to company $y_i \in Y$ represented as a vector (see the section on Wasserstein distance). We refer to *k* as the number of elements in the vector. We want to find a *k* such that we have sufficient expressiveness in the feature space to make useful comparisons between objects in various dimensions of interest—we aim to find a good representation. In the limit, *k* is equal to the number of networks. The problem, however, is that it is computationally expensive and, moreover, unnecessary because it does not increase the prediction accuracy (SM: Figure S7 shows converging behavior for $k=64$ and $k=128$).

### Reference set selection method

We represented each company as a vector of dissimilarities with respect to other companies. Assumming that we have a *k*-dimensional vector representation, that implies that we must select *k* reference networks. The selection of the reference networks can impact the expressiveness of the similarity vector. If we take, for example, a $k=2$ dimensional vector, selecting two reference companies that are similar to each other, then this results effectively in a $k=1$ dimensional vector because the company *x* will be equally similar to both companies in the reference set. To avoid this, we therefore first computed the pairwise similarity between all pairs of networks. Then we applied a k-medoids algorithm to identify *k* clusters and selected the medoid as a reference network. This ensured that we selected networks that are not similar to each other. We used the k-medoids clustering algorithm implemented in Scikit-learn^45^ to find *k* optimal reference items. For the random selection we used a uniform selection probability to sample *k* networks from the total population.

## Supplementary Information

Supplementary Information.

## Data Availability

The data that support the findings of this study are available from KPMG but restrictions apply to the availability of these data, which were used under license for the current study, and so are not publicly available. Data are however available from the authors upon reasonable request and with permission of KPMG.

## Code availability

The code to reproduce the results for the synthetic data and public data is available on GitHub: https://github.com/boersmamarcel/graph-sim
