# Supplementary material for: Measure cross-sectoral structural similarities from financial networks
Source: Sci Rep. 2023 May 2;13:7124. doi: 10.1038/s41598-023-34034-w (PMC10153030; doi:10.1038/s41598-023-34034-w)
Supplement: Supplementary file 1 — Supplementary Information. [file 41598_2023_34034_MOESM1_ESM.pdf]

Supplementary materials

M. Boersma[1,2,*], J. Wolsink[1,2], S. Sourabh[1], L.A. Hoogduin[3], and D. Kandhai[1]

[1]Computational Science Lab, University of Amsterdam, Amsterdam, the Netherlands
[2]KPMG, Amstelveen, the Netherlands
[3]KPMG Global Solutions Group, Berlin, Germany
[*]m.boersma@uva.nl

Supplementary materials

These supplementary materials show the additional analyses we performed on the company dataset and describe our modifications of the WWL[1] implementation. For the sake of clarity, we use the term WWL Reference (WWLR) in these materials to refer to our modified implementation.

First, we discuss why and how we modified the WWL implementation, and then we compare the results of WWLR with WWL and other state-of-the-art network classification algorithms. We start by performing an experiment to validate the WWLR algorithm: classify different types of generated networks. We then compare the performance of WWLR on network classification tasks using publicly available benchmark datasets. And finally, we discuss the additional results we obtained in our main experiment on the company dataset.

WWL modifications

The networks in the company dataset often exceed the average networks found in public datasets by orders of magnitude. As a result, it is not feasible to directly apply the implementation of WWL. We therefore had to modify or extend three components: the network data structure, the histogram construction and the vector representation (see the section on similarity vector representation). Our modifications resulted in performance similar to that reported by Togninalli et al.[1], but with one significant difference: the modified version is able to process the larger networks from the company dataset.

For the network data structure, we used a sparse network representation to store the networks in memory. In the company dataset we have networks with up to 700k nodes. The implementation as provided by Togninalli et al. uses the adjacency matrix which requires $O(N^2)$ storage for N nodes. With the sparse network representation we were able to process these adjacency matrices efficiently. In addition, we modified the propagation step so that it works with sparse matrix representations.

Togninalli et al. searches for an optimal transport map between the set of nodes of two graphs, requiring a cost matrix that uses $O(N^2)$ memory. For large networks, this is not feasible. Instead of mapping the nodes directly, we grouped them by value. This results in a histogram of node values. We then calculated an optimal transport map between the two histograms of the networks. The number of bins in each histogram was set to respect the memory limits. As a result, we reduced the memory complexity from $O(N^2)$ to a fixed set of bins stored as a sparse histogram.

In summary, we modified the WWL algorithm so that it is able to process large networks. The sparse network representation, the sparse histogram and the reference set of networks all resulted in a smaller memory footprint compared to the original implementation and allowed us to apply the concept of WWL to larger networks in the company dataset. In the sections that follow we will reproduce the results as obtained by Togninalli et al. to confirm whether our modifications achieve similar performance, with a focus on the impact of representing the company as a similarity vector with respect to a subset of reference networks.

Synthetic networks

Our aim was to evaluate whether the vector representation as proposed in the method section captures the relevant aspects of the network that make them distinct. To examine this, we performed the following experiment: we generated distinct networks and classified them. We assumed that the WWLR method captures the relevant aspects of a network. As a result, the more similar two networks are, the smaller their distance in vector space. Consequently, we should be able to find a good classifier that can identify groups of networks that are similar.

We generated groups of networks with similar characteristics: networks with random, hub and power-law characteristics. The generated networks from the same class should be more similar than the networks from different classes. Below we discuss each of the categories and their characteristics.

The **Erdös-Rényi model**[2] is a random network model that models networks that emerge because nodes randomly connect. In a way, it is comparable to social networks that emerge because people meet at random. The generation process was simple: for each pair of nodes we generated a random number, and if the number exceeded threshold p we connected the nodes with an edge – a random link. This model is often used as a baseline, to study which emerging patterns in a network could also be caused by randomness, revealing that real networks often show more hubs and a power laws for their degree distribution[3]. Figure S1 shows some sample random networks.

The **Strogatz-Watts model**[4] generates networks that have more hubs. Hubs are more often observed in real networks than obtained in random networks. To generate networks that have hubs, the Strogatz-Watts model generates a k ring structure and rewires nodes with a probability β. Figure S2 shows some sample network models generated by the Watts-Strogatz model.

The last network generation model, the **Barabasi-Albert model**[5], does not randomly connect nodes, but uses two important aspects to generate networks: growth and preferential attachment. Each time we added a node (growth step), we connected it to m other nodes with a probability that is proportional to the number of edges of that node – new nodes prefer to connect to highly connected nodes. This dynamic approach results in the power-law degree distribution often observed in real networks[3]. Figure S3 shows some sample networks.

This experiment resulted in three network classes that are distinct because they are random, contain hubs, or power laws. We used the network generation models to generate a set of networks that we labelled as Erdös-Rényi, Strogatz-Watts, and Barabasi-Albert. We generated 50 networks for each class resulting in a total of 150 networks. We used the NetworkX library to generate the networks[6]. For this dataset we created a vector representation of each of the networks (see the section on method) and used this representation to classify the network. Table S1 column *Graph Type* shows the classifications scores for the three characteristic network classes. We were able to classify each of the networks with 100% accuracy. This suggests that the vector representation accurately captures the relevant aspects of the networks.

In the second experiment, instead of generating networks from different classes, we generated networks from the same class but with different generation parameter settings. For example, the Barabasi-Albert model with $m = 5, 10, 15$ settings. This helps us to understand how smooth the vector representation is, and to determine if we can use the vector representation to discriminate between networks that are almost similar. We used the following settings for the data generation:

- Erdös-Rényi: $p = 0.1, 0.4, 0.8$
- Watts-Strogatz: $k = 5, 10, 15$ and $\beta = 0.2, 0.4, 0.8$
- Barabasi-Albert: $m = 5, 10, 15$.

For each class, we generated a dataset of 150 graphs with 100 nodes, and another dataset with 1000 nodes, resulting in a total of 6 datasets with 150 graphs. Furthermore, the values of the nodes were randomly initialized between 0.1 and 100 sampled from an uniform distribution.

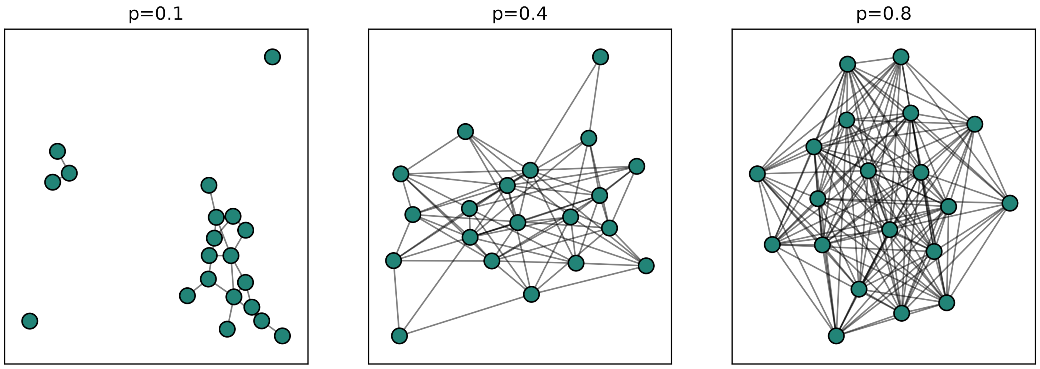

Figure S1. Three examples of the Erdös-Rényi model[2] with various parameters. The networks emerge from the random probability p to connect two nodes.

Table S1 the columns Erdös-Rényi, Watts-Strogatz and Barabasi-Albert, which show the prediction accuracy for the naive and WWLR algorithms. Again, we obtained a high accuracy score ranging from 99% to 100%. The two experiments show that we can accurately predict the classes of the network. Moreover, we can even predict within a single class the different parameters settings used, suggesting that the vector representation accurately captures the relevant aspects of the underlying network.

Table S1. Accuracy scores for the synthetic dataset for the naive classifier and the WWLR representations with the SVM classifier. The naive classifier predicts everything as the same (largest) class. We see that the WWLR representation can be used to accurately predict the classes.

Model & Graph type	Erdös-Renyi	Watts-Strogatz	Barabasi-Albert	Graph type
Naive	33.33 ± 0.0	33.33 ± 0.0	33.33 ± 0.0	33.33 ± 0.0
WWLR	100 ± 0.0	100 ± 0.0	99.33 ± 2.0	99.33 ± 2.0

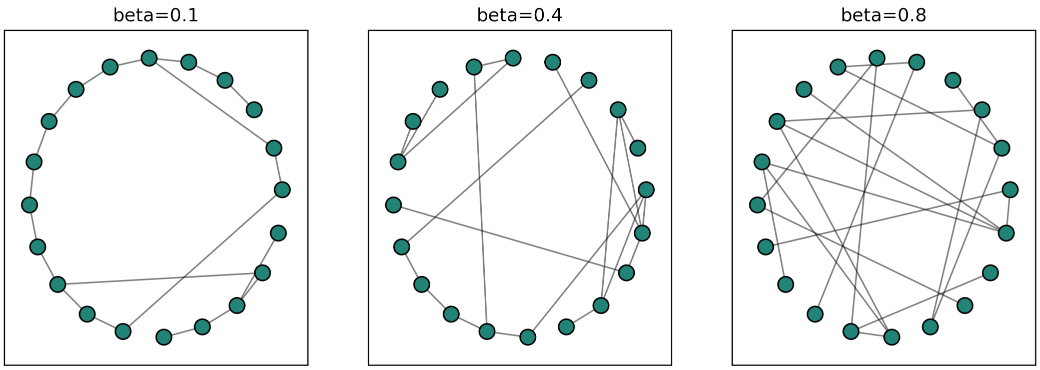

Figure S2. Three examples of the Watts-Strogatz model[4] with various parameters. The networks emerge from a ring structure that is randomly rewired.

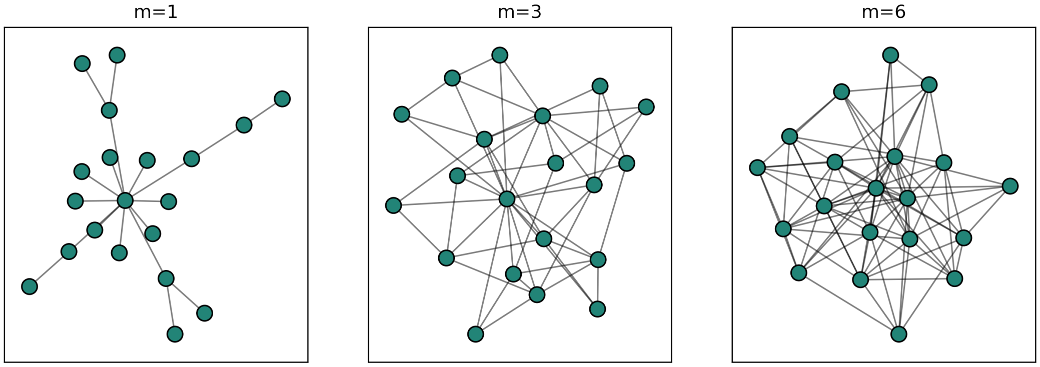

Figure S3. Three examples of the Barbabási-Albert model[5] with various parameters. The networks emerge by adding new nodes which have a preference to connect to highly connected nodes.

Public networks

The performance on synthetic networks suggests that the vector representations capture the relevant aspects. Yet, tests on real datasets give us more insights into the performance of the network classification algorithm. Therefore, we benchmarked the WWLR algorithm on publicly available datasets and compared its performance with other state-of-the-art algorithms. Table S2 shows the classification accuracy for several models and multiple public benchmark datasets. We found that WWLR performs on par with other algorithms and that our modification yields similar accuracy scores compared to WWL except for the ENZYMES dataset. Moreover, Table S3 shows the classification accuracy of the WWL[1] and the WWLR algorithms. We repeated the experiments of Togninalli et al.[1] to obtain new standard deviations that are better comparable with our results, and we discovered that the results are also better comparable with other public results (see Table S2). Togninalli et al.[1] splits the dataset into 10-folds: for each fold it reports the accuracy with the best parameters it could find for that fold[7]. The mean accuracy and the standard deviation are reported over the 10-folds. As a result, the standard deviations are low. We calculated the mean and standard deviation for a single parameter setting over 10 repetitions, meaning that we did not search for the best parameters within a repetition, and this method resulted in larger standard deviations. When we repeated the experiment of WWL within the new setup, we obtained standard deviations that are closer to the standard deviation of WWLR. Moreover, we observed that the standard deviations are closer to the standard deviations reported by others, see Table S2. Furthermore, we investigated the impact of the selection algorithm on the reference networks. We used the k-medoids algorithm and a random selection. Table S4 shows the classification accuracies for the public datasets. The mean performance of k-medoids is similar to that of the random selection. Moreover, the standard deviation is similar to the one obtained with a random selection. However, the random selection algorithm is computationally more efficient than the k-medoids algorithm.

Table S2. This table shows the mean accuracy scores and standard deviation obtained for the public network datasets. We reported the scores of other algorithms in this table for convenience and cite the source. WWLR scores at par with results obtained from other models.

Model	MUTAG	PTC-MR	NCI1	D&D	PROTEINS (con)	IMDB-B	IMDB-M
Naive	66.48 ± 0.0	55.81 ± 0.0	50.0 ± 0.0	58.65 ± 0.0	59.65 ± 0.0	50.00 ± 0.0	33.33 ± 0.0
WWLR-K (svm)	89.85 ± 4.5	65.11 ± 6.32	77.57 ± 3.68	77.01 ± 3.78	75.02 ± 4.49	73.30 ± 4.1	51.73 ± 4.56
WWLR-R (svm)	89.85 ± 6.92	66.27 ± 5.95	77.44 ± 3.50	77.51 ± 4.29	75.02 ± 4.24	73.30 ± 3.98	51.80 ± 3.91
WWLR (automl)	86.2 ± 8.87	60.16 ± 6.15	76.20 ± 3.85	76.24 ± 4.54	73.85 ± 3.42	78 ± 3.03	62.47 ± 5.93
WWL[1]	87.27 ± 1.5	66.31 ± 1.21	85.75 ± 0.25	79.69 ± 0.5	77.91 ± 0.81	74.37 ± 0.83	-
WWL*	89.98 ± 5.08	66.55 ± 6.36	81.7 ± 3.96	79.63 ± 3.46	75.48 ± 3.05	74.60 ± 5.02	52.00 ± 3.72
GOT[8]	-	61.19	-	-	-	63.63	-
WEGL[9]	89.3 ± 6.6	67.5 ± 7.7	78.4 ± 1.6	-	76.4 ± 4.2	75.4 ± 5.0	52.3 ± 2.9
DDGK[10]	91.58 ± 6.74	63.14 ± 6.57	68.1 ± 2.3	83.14 ± 2.73	-	-	-

Table S3. This table shows the average ten-fold cross validation classification accuracy and standard deviation for the public dataset. We show that WWLR is at par with respect to WWL, whereas WWL is computationally more expensive.

	MUTAG	PTC-MR	NCI1	D&D	Proteins	IMDB-B	IMDB-M
WWLR (SVM)	89.85 ± 4.50	65.11 ± 6.32	77.57 ± 3.68	77.01 ± 3.78	75.02 ± 4.49	73.30 ± 4.10	51.73 ± 4.56
WWL[1]	87.27 ± 1.50	66.31 ± 1.21	85.75 ± 0.25	79.69 ± 0.50	77.91 ± 0.80	74.37 ± 0.83	-
WWL (ours)	89.98 ± 5.08	66.55 ± 6.36	81.70 ± 3.96	79.63 ± 3.46	75.48 ± 3.05	74.60 ± 5.02	52.00 ± 3.72

Extended evaluation of the company dataset

In addition to the accuracy scores of the classification task in the company dataset, we investigated the vector representations from a qualitative point of view. We expected to find that neighbours in the vector space are related companies, and that companies from the same industry cluster together. We plotted the vector representations by using t-sne[11] to visually represent the high-dimensional vectors as a point in a 2-dimensional space. We expected to see cluster formation for companies from the same industry. Moreover, we selected a *retail company* and an *insurance company* and for both we selected the nearest neighbours and inspected these.

Figure S4 shows the t-sne visualization of the detailed industry dataset (151 companies), where each colour corresponds to an industry category. The companies from the *LE*, *PF* and *HLP* industries cluster together while *RTL* and *CRS* are more dispersed. A plausible reason for this is that auditors often assign *CRS* as a general industry for companies that are difficult to classify – for example, a diversified company which is economically active in a variety of industries cannot be classified into a single industry.

In Figure S5, we highlighted the nearest neighbours of the *retail company* and the *insurance company*. For the first cluster, the *retail company*, we found that 7 out of 10 are subsidiaries of the same company but from different geographic locations. Interestingly, if we were to order the companies in terms of geographic distance, we would more or less obtain the same order as when we order their vector distances. In this case, Belgium, France, Germany and then the Scandinavian countries. For the second cluster, the *insurance company*, we found that 8 out of 10 neighbours are also insurance companies and that the two with the smallest distance are subsidiaries of the same insurance company.

In Figure S6 shows our selection of two parent companies and their subsidiaries. Interestingly, for company A, two clusters emerged with a reasonable explanation. The top-left cluster (Orange) represents the European subsidiaries, whereas the cluster in the middle represents the Scandinavian subsidiaries. One explanation could be that accounting structures differ in

Table S4. This table shows the average ten-fold cross validation classification accuracy and standard deviation. The results indicate that random reference nodes yield the same prediction accuracy but are computationally more efficient.

Model	MUTAG	PTC-MR	NCI1	D&D	ENZ	PROT	IMDB-B	IMDB-M
K-mediods	89.85 ± 4.50	65.11 ± 6.32	77.57 ± 3.68	77.01 ± 3.78	47.67 ± 8.07	75.65 ± 3.69	73.3 ± 4.10	51.73 ± 4.56
Random	89.85 ± 6.92	66.27 ± 5.95	77.44 ± 3.50	77.51 ± 4.29	45.17 ± 7.17	75.02 ± 4.24	73.30 ± 3.98	51.80 ± 3.91

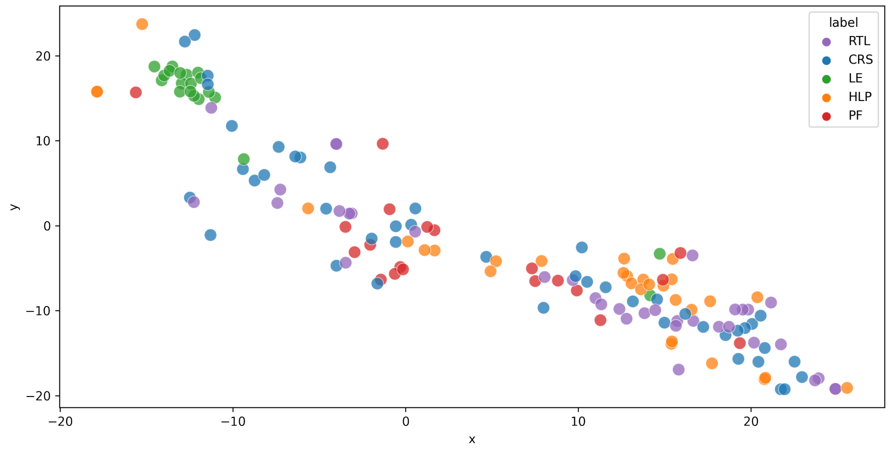

Figure S4. The t-sne visualisation where each item represents a company and the coluors of the items group them in one of the 5 industries. For some industries the companies cluster (LE, HLP, PF), whereas for others they are more scattered (RTL, CRS).

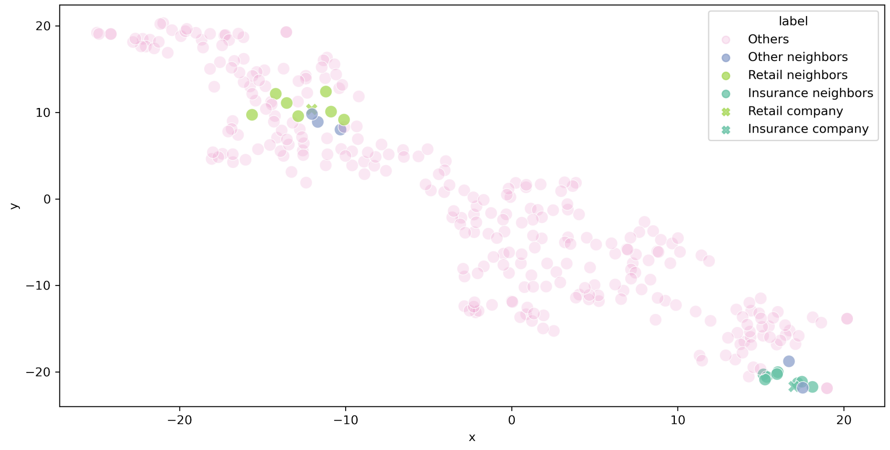

Figure S5. We selected a *retail* and an *insurance* company. For each company, we highlighted the nearest neighbours.

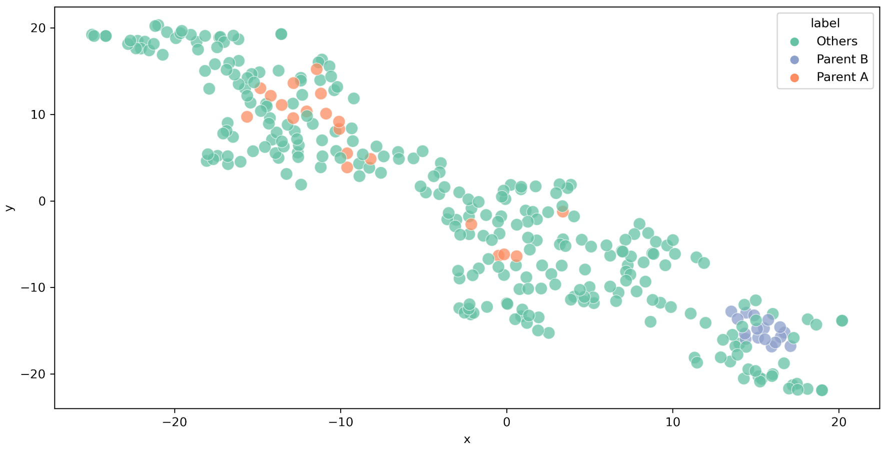

Figure S6. This figure displays two clusters of subsidiaries from company A and B as a t-sne visualization. Parent A consists of two clusters: one from central Europe and one from Scandinavia. Parent B consist of a single cluster.

Scandinavian countries compared with other European countries, another plausible explanation is that Scandinavian subsidiaries have less revenue and therefore cluster together. For parent B, we observed that all subsidiaries clustered together.

In summary, the vector representations and the distances between them result in clusters in line with our expectations. Subsidiaries of a company are close to one another, as are companies from the same industry. The distance between the vectors seems to capture the relatedness between two companies.

Sensitivity analysis

We investigated the impact on the accuracy scores for the parameters of the WWLR algorithm: the number of Weisfeiler-Lehman iterations and the number of reference networks. We performed a grid search over the parameter space and repeated each experiment 10 times to obtain a standard deviation. We performed the grid search for $h = 0, \ldots, 7$ and $r = \{2^n | n \in 1, 2, \ldots, 6\}$ where h is the number of iterations for the Weisfeiler-Lehman propagation step and r is the number of reference networks we used to create the vector representation. In the main text of this article we reported the accuracy score for the best performing parameter settings. Figure S7 shows the accuracy scores obtained for different settings of h and r. The gradient in colour for the number of iterations h suggests that increasing the number of iterations improves the classification accuracy. Similarly, the gradient in colour on the References r axis suggests that increasing the number of reference networks improves the classification accuracy. However, the increase in accuracy seems to be small for a large number of reference networks. This suggests that we can accurately describe a company with a subset of reference networks.

Table S5 shows our examination of the impact of the classification algorithm: SVM and the AutoML framework. For the AutoML framework we set the search time to 30 minutes to find a model and parameter setting that results in the highest accuracy scores. The AutoML framework results in lower scores for the company dataset. A possible explanation for this could be the large search space and the relatively short time to search for good models. Interestingly, for the public dataset (Table S2), we obtained significantly higher scores for the IMDB Binary and Multi dataset.

Table S5. This table shows the average ten-fold cross validation classification accuracy and standard deviation. We observe the impact of different classification models and see that the SVM classifier yields the highest scores.

Model	Industry	Industry detailed	International	Large balance
SVM	64.89 ± 10.56	74.83 ± 11.46	77.29 ± 5.01	76.65 ± 7.41
AutoML classifier	58.08 ± 8.29	65.58 ± 14.78	72.80 ± 5.11	68.89 ± 6.21

Analysis of the confusion matrix

The confusion matrix summarizes the algorithm's performance on a test dataset. We use the confusion matrix to understand the strengths and weaknesses of the algorithm. In the confusion matrix we show the predicted classes as columns and the actual

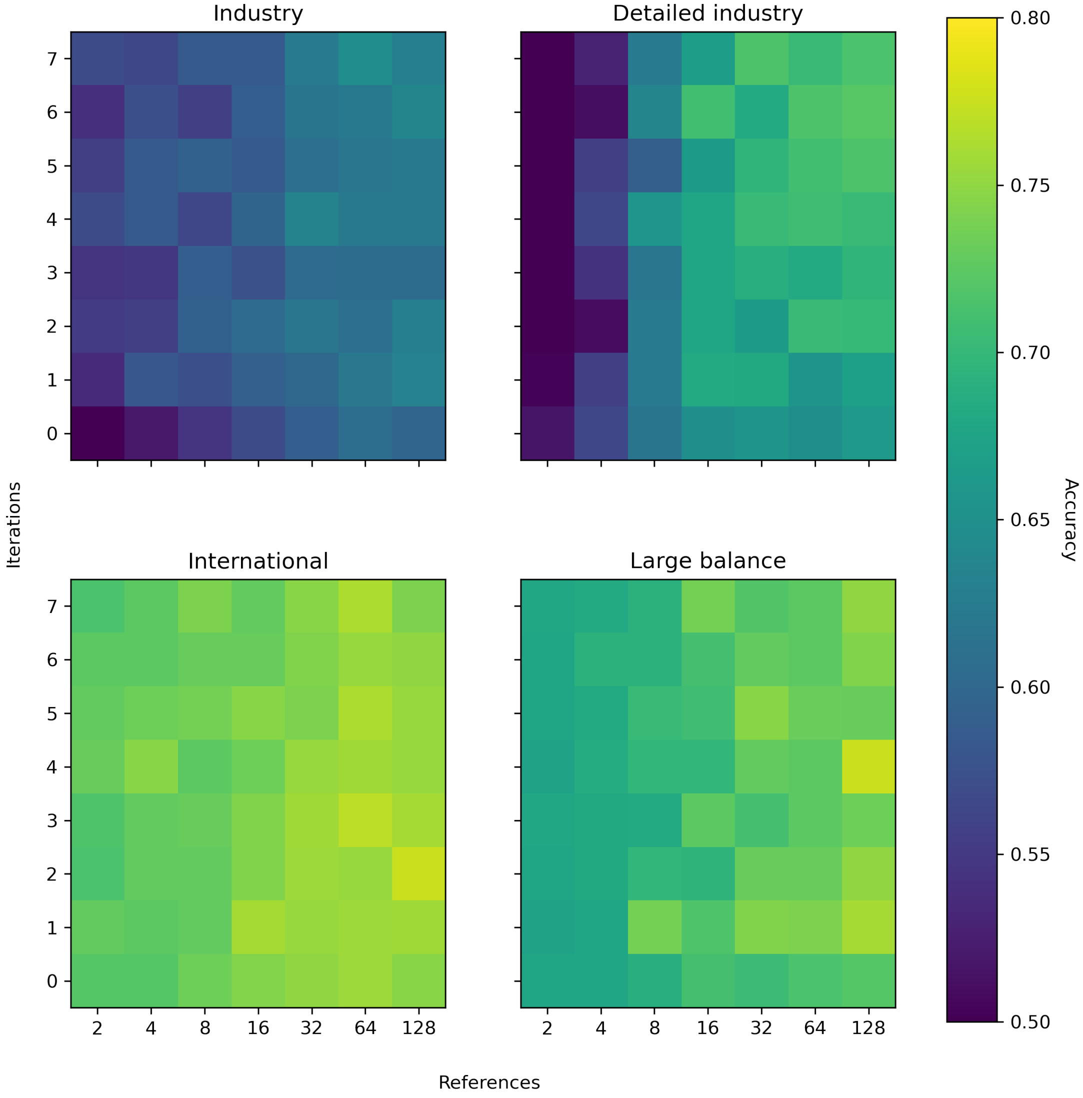

Figure S7. The accuracy scores as a function of the number of reference networks and the number of Weisfeiler-Lehman iterations.

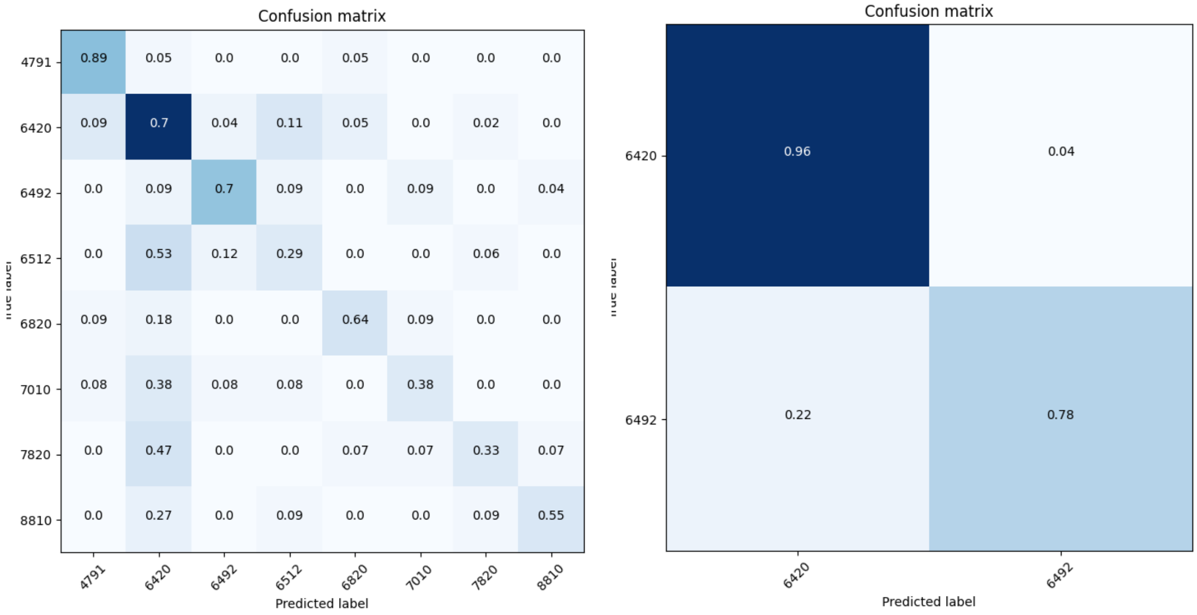

Figure S8. SBI/NACE class with on the left the confusion matrix for 10 items per class and on the right the confusion matrix for 20 items per class.

classes as rows. Note that we normalize the results per row. Therefore, we obtain a distribution over the predicted classes that sum to 1. Consequently, we can learn, for example, that the algorithm confuses a financial holding for a non-financial holding or vice versa. In this section, we show and discuss the confusion matrix for several classifications to evaluate the performance.

For the NACE industry classes, we show two confusion matrices in Figure S8 with on the left the confusion matrix when we have at least 10 items per class and on the right for 20 items per class. Note that, for 20 items per class, we have only two classes: Activities of holding companies (6420), and Other credit granting (6492). The accuracy is high at 91% and the confusion matrix shows that for 22% of the cases, it wrongly predicts another class – it classifies Other credit granting (6492) as a Activities of holding companies. The other way around only occurs 4% of the time. For the 10 items per class, we observe that we have 8 classes in total. The performance per class ranges from 29% up to 89%. Interestingly, we often mistake a class to be an Activities of holding companies class (6420). A possible explanation is, in the SBI/NACE classification, we often notice that a company is classified as an Activities of holding companies (6420) but it could be a holding active in various industries, for example, a holding in the retail industry or a holding in the banking industry. Consequently, the algorithm confuses the other classes as holding classes, especially, because the holding class is also the largest in the dataset. Interestingly, the class Activities of holding companies (6420) and Activities of head offices (7010) are often mistaken. Not surprisingly, the results suggest that there are not that many structural differences between the two classes. In the case of multiple SBI/NACE classes for a company, we selected the most appropriate label. When we select, however, the first label we notice a drop in performance for the 20-items-per-class from 91% to 77%. For the 10 items per class, we have the same accuracy.

For the audit expert's classification, we observe in Figure S9 that we have a true positive percentage between 0% and 80%. Interestingly, whenever the algorithm makes a classification error, it often confuses it to be a *General manufacturing* (CRS) company. A possible explanation is that CRS is the largest class in the dataset and the algorithm overfits towards the largest class. Moreover, the variety of companies assigned to the CRS class is larger than other classes. Therefore, it might be more difficult to find the structural characteristics of that industry.

Sensitivity: items per class

In our dataset we have many classes as a result most classes have only a few items. To evaluate the performance of our algorithm, we select classes with sufficient items. Therefore, we investigate the sensitivity of this parameter, we set it to 10 and 20 and study the impact thereof on the accuracy. Trivially, the lower the number of items required, the more classes we have. Consequently, with fewer items per class, it is more challenging for the algorithm to learn how to classify them. Therefore, we expect lower levels of accuracy compared to fewer classes with more items per class.

Our results confirm this finding, we show the accuracy and confusion matrices for the 10 items per class and 20 items per class. First of all, we notice an increase in accuracy when the number of samples per class increases – the algorithm is better

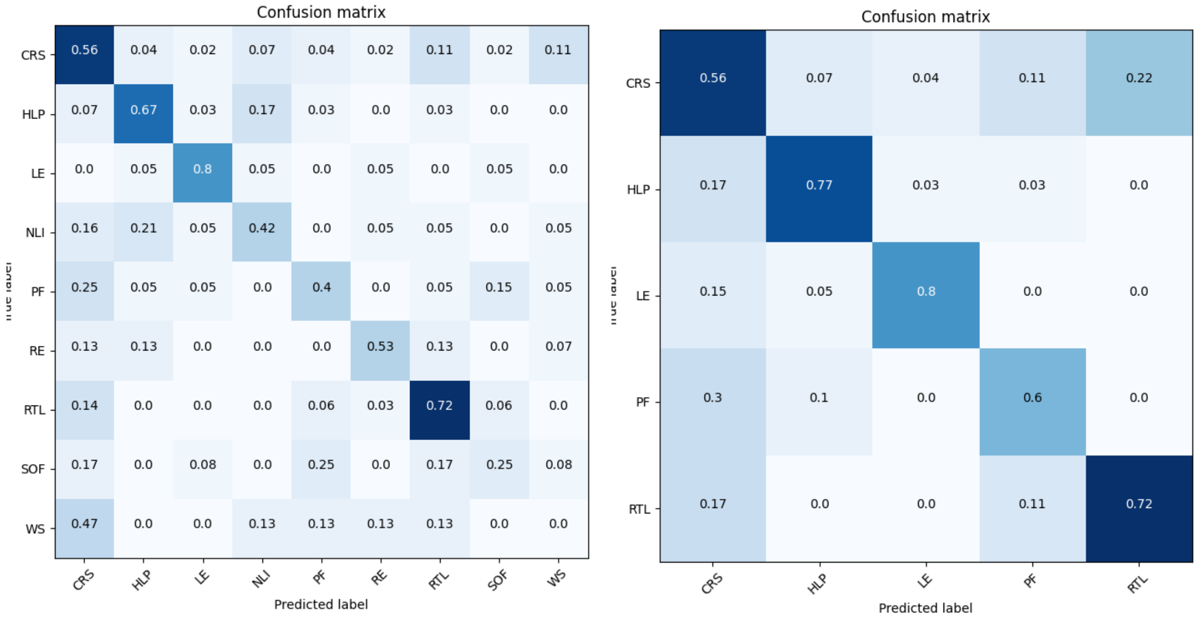

Figure S9. Detailed industry expert classes with on the left the confusion matrix for 10 items per class, and on the right the confusion matrix for 20 items per class.

able to learn the structural characteristics of the network. Moreover, when there is less noise (classes with fewer samples), the algorithm improves. For example, in Figure S9, we notice that some classes like *Publishing* (PF) have good performance for the 20 items per class, but less for the 10 items per class. We see that it confuses the PF with the *Software* (SOF) industry. Also, the *Whole sale* (WS) industry is not recognized at all and is spread evenly amongst 13% *Non-life insurance* (NLI), 13% *Publishing* (PF), 13% *Real estate* (RE), 13% *Retail* (RTL), and 47% *General manufacturing* (CRS).

Table S6. This table shows the accuracy scores when changing the sufficient samples per class criteria between 10 and 20.

Class type	10-items	20-items
SBI/NACE	61%	91%
Industry Detailed (expert)	55%	75%
Industry (expert)	53%	66%

Table S7. This table shows the total sample size when changing the sufficient samples per class criteria between 10 and 20.

Class type	10-items	20-items
SBI/NACE	166	80
Industry Detailed (expert)	212	151
Industry (expert)	285	246

Manipulation detection

We used the vector representation of a company to classify to which industry it belongs. This is a good test case to validate whether the vector representation captures the relevant aspects of the network. But from an audit perspective, it is even more interesting to see the other possibilities that a good vector representation provides: it can detect significant changes in the network structure that identify potentially higher audit risks.

In our experiment, we selected a set of companies with multiple years of data and measured the network similarity between years. We used this similarity as a baseline to detect significant changes in the network structure. We selected three company datasets that had two consecutive years of transaction data – we only had a limited number of datasets available that satisfied

the multi-year criteria. For each company, we determined their vector representations for 2019 and 2020, and measured the distance between the two vector representations. The dissimilarity between two consecutive years represents differences that occur due to the company's natural evolution. In addition, we generated a modified dataset. We modified the 2020 accounting data by selecting another company and mixing the accounting entries between the two companies equally (50-50). We used the cosine similarity to measure the similarity between two vectors because we merged two accounting systems that might differ in size and we only wanted to measure the structural difference. In the experiment, we wanted to find a lower cosine similarity between the 2019 data and the mixed data compared to the 2019 and 2020 data of the same company. The mixed company is referred to as Company AB.

For experiment 1, we obtained a cosine similarity of 0.97307113 between the vectors of company A in 2019 and 2020. When we compared this with the modified data, we obtained a cosine similarity between company A in 2019 and company AB of 0.9062461 which is significantly lower. For experiment 2, we obtained a cosine similarity of 0.99955081 between consecutive years and a cosine similarity of 0.98020302 between 2019 and the modified dataset. Interestingly, we selected company C from the same industry. As a result, the difference in cosine similarity is still measurable but smaller compared to experiment 1 and 3 which use a company C from a different industry. For experiment 3, we obtained a cosine similarity of 0.97307113 between consecutive years and 0.9062461 between 2019 and the modified dataset. In all cases we found that the cosine similarity was lower for the modified dataset. The results suggest that we can indeed use the network similarity to detect significant changes in accounting structure, identifying potential changes in audit risk.

References

1. Togninalli, M., Ghisu, E., Llinares-López, F., Rieck, B. & Borgwardt, K. Wasserstein weisfeiler-lehman graph kernels. *arXiv preprint arXiv:1906.01277* (2019).
2. Erdős, P. & Rényi, A. On the evolution of random graphs. *Publ. Math. Inst. Hung. Acad. Sci* **5**, 17–60 (1960).
3. Barabási, A.-L. Network science. *Philos. Transactions Royal Soc. A: Math. Phys. Eng. Sci.* **371**, 20120375 (2013).
4. Watts, D. J. & Strogatz, S. H. Collective dynamics of 'small-world'networks. *nature* **393**, 440–442 (1998).
5. Barabási, A.-L. & Albert, R. Emergence of scaling in random networks. *science* **286**, 509–512 (1999).
6. Hagberg, A. A., Schult, D. A. & Swart, P. J. Exploring network structure, dynamics, and function using networkx. In Varoquaux, G., Vaught, T. & Millman, J. (eds.) *Proceedings of the 7th Python in Science Conference*, 11 – 15 (Pasadena, CA USA, 2008).
7. BorgwardtLab. Borgwardtlab/wwl (2019).
8. Maretic, H. P., Gheche, M. E., Chierchia, G. & Frossard, P. Got: An optimal transport framework for graph comparison. *arXiv preprint arXiv:1906.02085* (2019).
9. Kolouri, S., Naderializadeh, N., Rohde, G. K. & Hoffmann, H. Wasserstein embedding for graph learning. *arXiv preprint arXiv:2006.09430* (2020).
10. Al-Rfou, R., Perozzi, B. & Zelle, D. Ddgk: Learning graph representations for deep divergence graph kernels. In *The World Wide Web Conference*, 37–48 (2019).
11. van der Maaten, L. & Hinton, G. Visualizing Data using t-SNE Laurens. *J. Mach. Learn. Res.* **9**, 2579–2605, DOI: 10.1007/s10479-011-0841-3 (2008).
